# Novel Anti-Melanoma Immunotherapies: Disarming Tumor Escape Mechanisms

**DOI:** 10.1155/2012/818214

**Published:** 2012-04-23

**Authors:** Sivan Sapoznik, Ohad Hammer, Rona Ortenberg, Michal J. Besser, Tehila Ben-Moshe, Jacob Schachter, Gal Markel

**Affiliations:** ^1^Ella Institute for Melanoma, Sheba Medical Center, Ramat Gan 52621, Israel; ^2^Pontifax Venture, Herzliya, Israel; ^3^Department of Clinical Microbiology and Immunology, Sackler Faculty of Medicine, Tel Aviv University, Ramat Aviv 61390, Israel; ^4^cCAM Biotherapeutics Ltd., Kiryat Shmona 11013, Israel; ^5^Talpiot Medical Leadership Program, Sheba Medical Center, Ramat-Gan 52621, Israel

## Abstract

The immune system fights cancer and sometimes temporarily eliminates it or reaches an equilibrium stage of tumor growth. However, continuous immunological pressure also selects poorly immunogenic tumor variants that eventually escape the immune control system. Here, we focus on metastatic melanoma, a highly immunogenic tumor, and on anti-melanoma immunotherapies, which recently, especially following the FDA approval of Ipilimumab, gained interest from drug development companies. We describe new immunomodulatory approaches currently in the development pipeline, focus on the novel CEACAM1 immune checkpoint, and compare its potential to the extensively described targets, CTLA4 and PD1. This paper combines multi-disciplinary approaches and describes anti-melanoma immunotherapies from molecular, medical, and business angles.

## 1. Introduction

The interplay between cancer cells and the host immune system displays an intriguing, dynamic battle for life. The current dogma on tumor progression under immune pressure is of the three “E”s: elimination, equilibrium, and escape [1]. In the first phase, the innate and adaptive immune system tracks and eliminates nascent tumor cells (immune surveillance). If not all cancer cells are eliminated, the second phase is equilibrium between cancer and the immune system, in which for a while, sometimes lasting years, the tumor remains dormant. This equilibrium, however, is temporary as genetic instability of cancerous cells together with continuous pressure of immune cells gradually shapes the immunogenicity of the tumor, transforming it into poorly immunogenic. This process, called immune editing, leads eventually to tumor escape and thereby progression into clinically evident disease. The immune system thus suppresses tumors on the one hand while promoting it on the other hand, by selecting and encouraging poorly-immunogenic variants (reviewed in [1–3]). The mechanisms of tumor escape are numerous. They include alteration of the features of the tumor cells themselves (up-regulation of anti-apoptotic molecules and of cytotoxic determinants and downregulation of antigen presentation MHC molecules), secretion of cytokines that inhibit effective immune response (e.g., VEGF, IL-10, and TGF*β*), and the induction of an immuno suppressive environment by indoleamine 2,3 dioxygenase (IDO) or via recruitment of inhibitory immune cells (Treg, MDSC, NKT, iDC, and macrophages) [4–6].

We will focus here on metastatic melanoma, which is an excellent example for the above mentioned model, as it is highly immunogenic and responds to immunotherapy [7]. Malignant melanoma is a main cancer-related cause of death in people below thirty. It is the most rapidly increasing malignancy in Western population in terms of incidence and is currently the sixth most common cancer in the USA, displaying high mortality rate, surpassed only by lung cancer [8, 9]. As surgery is beneficial only for localized (primary) melanoma, continuous efforts are made to find effective immunotherapies for metastatic melanoma (MM). Systemic treatments include the administration of nonspecific immune-stimulating cytokines [7], immunization with cancer cells or molecules [10], adoptive T cell transfer [11], the recently developed small inhibitors of melanoma oncogenes [12], and blocking antibodies against inhibitory immune molecules [13]. Accumulating data proved that melanoma induces both innate and adaptive immune responses and that immune cells home to and infiltrate melanoma masses. However, the avidity of these cells is probably low, due to low cell number, low cytotoxic potential, or inhibitory microenvironment [14–17]. We will here describe promising immune treatments that aim to enhance the naturally occurring anti-melanoma immune response.

## 2. Anti-Melanoma Immunotherapies

### 2.1. Anti-CTLA4 (Ipilimumab and Tremelimumab)

CTLA-4 is an inhibitory molecule expressed on T cells undergoing activation, which functions to prevent prolonged activation signals. T cells are activated by two sequential signals: antigen recognition (TCR binding to antigen/MHC on APCs) and costimulation (e.g., CD28 interaction with B7.1 or B7.2 on APCs). CTLA-4 competes with CD28 on the binding of B7 and, when upregulated, inhibits CD28-dependent proliferation and activation and instead leads to cell cycle arrest, decreased cytokine production, and IDO secretion from APCs [18, 19]. Noteworthy, it was reported that CTLA-4 is also expressed by various tumor cells [20] and in a Wnt-dependent manner in melanoma [21]. Stimulation of tumor-expressed CTLA-4 with soluble ligands or agonistic mAb leads to induction of apoptosis [20, 21] as well as inhibition of proliferation and secretion of angiogenic cytokines [22]. These observations point out that CTLA-4 exerts nonimmune-related functions when expressed by nonlymphoid cells. It could also reflect a yet undefined mechanism by which tumors achieve an “immune escape” phenotype and actively suppress, evade, and avoid T cell immunity [23].

Complete knockout of CTLA-4 is lethal, and mice suffer from massive lymphoproliferation and organ destruction [24, 25]. However, preclinical studies showed that blocking of CTLA-4 results in anti-tumor activity and tumor regression in many mice tumor models (prostate, breast, lymphoma, melanoma) [26–29], which paved the way for clinical studies. Two anti-CTLA-4 monoclonal antibodies, generated by different companies, were tested in clinical trials in MM patients: Tremelimumab (Pfizer) and Ipilimumab/Yervoy (Bristol Myers Squibb), but only the latter was successful in phase III studies. Based on its ability to prolong survival of previously treated as well as untreated MM patients [30, 31], Ipilimumab gained European Union (2010) [32] and FDA (March 2011) approval.

Two exciting phase III studies tested the clinical effects of Ipilimumab in advanced MM patients. In the first, 676 participants from 125 different medical centers that were already treated with standard treatments received either Ipilimumab, gp100 vaccine, or the combination of both, in a randomized, double-blind manner. Treatment with Ipilimumab improved median overall survival rates (10.0 and 10.1 months in the Ipilimumab-treated groups as compared with 6.4 months in the gp100-only treated group). The percentages of the patients who responded to Ipilimumab in the two groups were very limited (complete response in ~1% and partial response in 5–10%), but the effects of response were long-lasting in the majority of the responders [30]. In the second trial [31], 502 patients that were not previously treated received either dacarbazine (DTIC, standard care chemotherapy) or Ipilimumab in combination with dacarbazine in a double-blind, placebo-controlled manner. In this experiment, Ipilimumab increased overall survival rates from 9.1 to 11.2 months and 3-year survival from 12.2% to 20.8%. Adverse effects, mainly immune related in the skin and gastro-intestinal track, accompanied nearly all patients in the two trials, with about half of the patients suffering from severe adverse effects in the second trial and several severe immune effects-related deaths in the first trial. These exciting results thus also exhibit the complicity of specifically manipulating immune responses.

### 2.2. Anti-PD1 (MDX-1106 and CT-1101)

PD-1, as CTLA-4, is an inhibitory receptor belonging to the CD28 superfamily of immune-regulatory receptors. However, while CTLA-4 expression is limited to T cells, PD-1 has a broader expression profile and is expressed on activated T, B and several myeloid cells. PD-1 (programmed death 1) downregulates T cell function (proliferation, cytokine secretion, and cytolysis of target cells) by delivering negative signals upon binding to its ligands, PD-L1 and PD-L2 (reviewed in [33]). PD-L2 expression is restricted to APCs (dendritic cells and monocytes) [34–36], and it is involved in tolerance of T cells to environmental (e.g., orally administrated) antigens [37]. PD-L1, on the contrary, is expressed by multiple normal and cancerous tissues and confers peripheral tolerance from “self” antigens [38, 39]. Upon normal levels of antigen exposure, PD-1 functions as a “gate keeper” to attenuate immune responses (reviewed in [40]). The importance of PD-1 is manifested in PD-1-deficient mice, which suffer from auto-immunities [41, 42]. Upon abnormal antigen exposure levels (chronic viral infection, caner) however, this immune tolerance becomes a stumbling block, as PD-1 delivers “veto” signals for CTLs, a response which renders tumor cells protected from cytotoxic immune cells and hampers anti-tumor immune interventions, such as vaccinations and ACT [40]. PD-L1 is upregulated in cancerous cells *in vitro* by immune cytokines, which are critical for T cell functioning, such as IFN*γ* [43], which may even positively feedback to enhance immune tolerance *in vivo*. Indeed, PD1-deficient mice exhibit enhanced anti-tumor T cell responses towards solid and hematopoietic tumor, including melanoma, these mice survive longer and the tumors are regressed [39, 44, 45] and tumor transduced to overexpress PD-L1 grew more aggressively *in vivo* [46]. Blocking the PD1/PD-L1 pathway delays tumor progression [39, 44, 47–49] and adoptive transfer of tumor-specific PD-1-deficient T cell receptor transgenic T cells can reject tumors [43]. In melanoma patients, PD-L1 is expressed on melanoma cells and the levels of PD-L1 expression positively correlate with overall survival [50]. PD-1 is upregulated in CD-8^+^ T cells from melanoma patients during the metastatic (III, IV) stages of disease [50] and this upregulation may be associated with T cell dysfunction [51]. 

In order to block the inhibitory PD-1/PD-L1 pathway, two different anti-PD-1 monoclonal inhibitory antibodies were generated, MDX-1106 (BMS-936558) [52] and CT-011 [53]. Phase I clinical studies with each of the antibodies proved their safety, well-tolerated administration, and limited toxicity (though in both of them the maximum tolerated dose was not reached) and provided pharmacokinetic data [52, 53]. In these clinical experiments, MDX-1106 (fully human antibody) was assayed in 39 patients with advanced melanoma, colorectal cancer, prostate cancer, non-small-cell lung cancer and renal cell carcinoma [52]. In the CT-011 study (humanized antibody), 17 patients were included, with leukemia, lymphoma, or multiple myeloma [53]. Clinical benefit was observed in both experiments [52, 53] and clinical responses correlated with the extent of PD-L1 expression on tumors [52]. Phase II clinical studies with MDX-1106 are ongoing with biweekly administration in metastatic non-small-cell lung cancer, renal cell carcinoma, prostate cancer and metastatic melanoma. They show limited toxicity, good tolerance (maximum tolerated dose (MTD) was not reached) and anti-tumor activity with 37.5% objective response in the total patients cohort (including 3 melanoma patients). One of the most impressive results was that all responses were highly durable and were still ongoing when publishing these preliminary results [54]. Phase II clinical trials with CT-011 are also ongoing (http://www.clinicaltrials.gov/). Two other antibodies of the PD-1 pathway are under clinical development (currently recruiting participants for phase I studies): MK-3475 (anti-PD-1) and MDX-1105-01 (anti PD-L1) (http://www.clinicaltrials.gov/). The combination of anti-PD-1 and anti-CTLA-4 was tested in murine B16 melanoma model and found to be more effective in tumor regression as compared to each of the blocking antibodies alone [55]. A phase I clinical trial involving the two antibodies is ongoing, as well as a trial that combines MD-1106 with melanoma vaccines (http://www.clinicaltrials.gov/).

### 2.3. Comparison between Anti-CTLA-4 and Anti-PD-1

**Figure 1:**
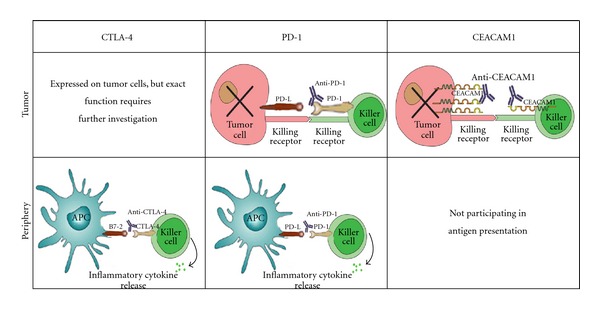
Sites of action for selected immune modulators. Anti-CTLA-4 targets the interactions between T cells and APCs in the periphery and thus is prone to evoke general stimulation accompanied by frequent adverse effects. Anti-PD-1 may block peripheral interactions, as anti-CTLA-4, but also the interactions between tumors and infiltrating immune killer cells, and thus may evoke both general and localized stimulation. CEACAM1 is expressed both on immune killer cells and on the tumor target, and anti-CEACAM1 is expected to act specifically at the interface between these two and to evoke low autoimmunity.

The different features of CTLA-4- as compared with PD-1-deficient mice [25, 42] and the synergism of anti-CTLA-4 and anti-PD-1 treatment in animal models [55] suggest that they act in distinct, non-redundant pathways. Though not enough experimental data using anti-PD-1 has been collected, the MTD of anti-PD-1 was not yet reached, and the drugs were not compared in a randomized manner, anti-PD-1 seems to evoke less severe and less frequent adverse effects as compared with anti-CTLA-4 [52]. These differences may be attributed to the different cellular targets of the drugs. Anti-CTA-4 targets a peripheral interaction, between T cells and APCs. Thus, it is expected to cause general stimulation accompanied by adverse effects. The exact mechanism of action of MDX-1106 is not known. However, as it blocks the interactions of PD-1 with both PD-L1 and PD-L2 [52], it may act not only in the periphery but also within the tumor sites, interfering with T cell/tumor cell interactions and evoking specific, localized stimulation. In searching for localized immune modulators, which act within the tumor milieu and whose manipulation will not lead to severe autoimmunity, we have studied the roles of CEACAM1 in melanoma (Figure 1).

### 2.4. CEACAM1 as a Novel Immunotherapeutic Target

Carcinoembryonic antigen-related cell adhesion molecule 1 (CEACAM1, CD66a), a member of the Ig superfamily, is a broadly expressed, multifunctional, cell-cell adhesion molecule [56, 57]. While not expressed in normal melanocytes [58], it is neoexpressed by the vast majority of melanoma specimens (unpublished observation) and is elevated during the histopathological progression of metastatic melanoma [59]. CEACAM1 is considered as an independent, highly significant marker for the development of melanoma metastases and poor survival [60]. Accumulating *in vitro* evidence suggests that it is not merely a marker but also confers cancerous characteristics to melanoma cells and thus may actively participate in the etiology of melanoma [58, 61]. In the immune system, CEACAM1 acts as an inhibitory molecule that blocks proliferation and cytotoxic activity of T cells [62–64] and NK cells [62, 65–70] via ITIM sequences and the recruitment of SHP-1 and SHP-2 phosphatases [69, 71, 72]. Supporting this immune-inhibitory role, the expression of CEACAM1 on target cells, including melanoma, protects them from being eliminated *in vitro* by NK and T cells [62, 64, 69]. We have recently reported that melanoma cells that have survived an *in-vitro* T cell attack actively increase CEACAM1 expression in an IFN*γ*-dependent manner [64] and that this elevation enhances the protective effect against subsequent immune attacks [63]. Moreover, we could identify CEACAM1-positive NK cells in lymph nodes infiltrated with CEACAM1-positive melanoma cells, but not with CEACAM1-negative melanoma cells [69]. These data suggest a potentially novel tumor escape mechanism that could be used by CEACAM1-positive melanoma cells to evade elimination by transferring CEACAM1 to the attacking immune cells. Indeed, transfer of CEACAM1 was observed *in-vitro*, although it was considerably less efficient than transfer of CEACAM5 [73]. Importantly, patient-derived melanoma infiltrating lymphocytes [64] and circulating T and NK cells from melanoma patients [68] synthesize and express functional CEACAM1 [64, 68, 69, 74], which renders them susceptible to CEACAM1-mediated inhibition and may thus contribute to cancer progression. We have observed over-expression of CEACAM1 by circulating cytotoxic lymphocytes in other diseases, including ankylosing spondilitis and bare lymphocyte syndrome type I [65, 67, 68], as well as on decidual lymphocytes obtained from CMV-infected pregnancies [62], all occurring due to yet to be defined mechanisms. These may be related to aberrant immune stimulation or to abnormal development of immune cells [68].

Based on these findings, we have developed a high-affinity murine monoclonal antibody against human CEACAM1 [75]. Anti-CEACAM1 does not act on CEACACM1-positive cells in *cis* (i.e., does not interfere with general cellular processes such as proliferation and apoptosis). Rather, it acts in *trans*, binding both T cells and melanoma, to efficiently relieve the CEACAM1-dependent inhibition of T cell cytotoxicity. Therefore, the mechanism of action *in vivo* of anti-CEACAM1 strongly depends on the endogenous immune system and its ability to recognize the target cells in an antigen-restricted manner, thereby reducing the risk of adverse effects stemming from generalized non-specific immune stimulation. We showed that anti-CEACAM1 renders melanoma cells susceptible to elimination by T cells, both *in-vitro* and in a human-melanoma xenograft murine model, which maintains antigen-restricted recognition [75]. Indeed, we have previously shown that abolishment of CEACAM1 with polyclonal anti-CEACAM antibodies does not induce a nonspecific T cell function [64].

Several lines of evidence pointed to the potential high specificity of anti-CEACAM1 to the cancerous state and to its potentially low risk of evoking adverse effects: (a) staining of normal tissue micro-array with anti-CEACAM1 proved only limited staining of luminal cells of some secretory ducts. These patterns are substantially more restricted than staining patterns of other FDA-approved therapeutic antibodies, such as Erbitux; (b) the anti-CEACAM1 mAb does not elicit complement-dependent cytotoxic effect nor non-specific T cell activation; (c) the anti-CEACAM1 mAb is not an agonistic antibody and is therefore probably incapable of exerting direct functional effects on CEACAM1-positive cells. Rather, it is an antagonistic antibody, whose effects depend on antigenic recognition between T cells and their targets; (d) the immune-inhibitory homophilic CEACAM1 interactions are expected to take place only in the tumor, and not during earlier stages of the elicited immune response, such as antigen presentation. CEACAM1 homophilic interactions occur between CEACAM1-positive cancer cells and CEACAM1-positive tumor infiltrating lymphocytes, which are late-effector lymphocytes. Thus, blocking of CEACAM1 is expected to enhance the immune response only within tumor sites and only in the context of antigen-restricted recognition. These exciting results mark anti-CEACAM1 as a potential specific and safe (compartmentalized to the tumor vicinity) novel immunotherapeutic modality (Figure 1). Another important advantage of CEACAM1-directed therapy is that patient selection would be based on the presence of CEACAM1 on tumor tissue. It should be noted that CEACAM1 is expressed in 60–80% of metastatic melanoma cases, which suggests that the majority of metastatic melanoma patients would benefit from anti-CEACAM1 antibodies. The anti-CEACAM1 approach is developed by cCAM BioTherapeutics, and first-in-man clinical trials are anticipated in the near future.

### 2.5. Adoptive T Cell Transfer (ACT)

Adoptive cell therapy with *ex vivo* cultured T cells, developed by Rosenberg and his colleagues in the National Cancer Institute, is currently the most promising immunotherapy for MM patients, yielding 50–70% objective response rates [76, 77]. It is based on the isolation of bulk T cell masses from resected melanoma, their *ex-vivo* expansion by about 1000-fold (reaching about 50 × 10^9^ cells), and their reinfusion to the patient following lymphodepleting nonmyeloablative chemotherapy, which eliminates endogenous competitor immune cells [76, 77]. Recently, we have shown that T cells derived from enzymatic digestion of resected tumors (rather from multiple small fragments) yield high numbers in culture, which enable to shorten their *ex-vivo* culturing period [78]. We and others have shown that responding patients were treated with TIL that spent less time in culture [78, 79]. Indeed, Young TIL cultures were successfully established for nearly 90% of MM patients, and overall response rates reached 50% [78]. The main disadvantages of ACT are that the generation of TIL cultures presents a technical challenge and is labor, cost and time consuming [76, 77]. Attempts to overcome several of these limitations by the usage of genetically-engineered rather than endogenous T cells were presented in two clinical trials. These trials, in which T cells were modified to overexpress TCR directed against melanoma antigen (MART-1 or gp100), yielded modest response rates (12–30%) but proved the feasibility of the method [80, 81]. Engineering T cells with chimeric antigen receptors (CARs), which recognize tumor cells in a MHC-independent manner and endow increased T cell activity [82], have been tested in preclinical studies in melanoma [83]. Recently, Peng and his colleagues have shown that over-expression of the murine chemokine receptor CXCR2 on T cells improves their homing to melanoma and tumor regression in mice model [84], suggesting that endowing T cells with improved chemotaxis capabilities to tumor sites may also enhance ACT.

### 2.6. Additional Antibodies

Additional immune-modulatory molecules that have gained scientific attention and are now under clinical development are OX40 (CD134), CD40, GITR, and 4-1BB (CD137) (http://www.clinicaltrials.gov/) (Table 1, also reviewed in [85]).

## 3. Cancer Immunotherapy: Business Angle

For decades, cancer immunotherapy has been neglected by drug development companies, which were deterred by the field unfavorable track record. Moreover, in order to achieve clinical proof of concept, immunotherapy requires a unique development strategy that involves long-term follow-up and randomized controlled studies. This stems in part from the fact that tumor shrinkage is less common with immunotherapy drugs coupled with the long period required for mounting a systemic immune response. [86]. Lastly, combining immune-modulating drugs with chemotherapy regimens was perceived as counterproductive.

This sentiment has gradually been changing following clinical validation with cancer vaccines and immunomodulatory antibodies. Of particular importance were FDA approvals for Sipuleucel-T and Ipilimumab, based on survival benefit in prostate cancer and melanoma, respectively, [31, 87]. These agents demonstrated unequivocally the value of immunotherapy for cancer in broad unselected populations.

The renewed interest in cancer immunotherapy is best exemplified by recent deals involving clinical and preclinical programs. The growing number of transactions coupled with their lucrative financial terms serves as a testament to the excitement within the industry regarding harnessing the immune system to fight cancer. Although melanoma remains a common indication for immunotherapies, recent data clearly suggests that potential utility for this approach spans well beyond this indication.

## 4. Deals

In August 2010, Amplimmune licensed MP-224, an Fc-fused PD-L2, to GlaxoSmithKline (GSK). Amplimmune received an upfront payment of $23 M and is eligible to receive $485 M in milestone payments. MP-224 binds and inhibits the immunosuppressive activity of PD-1, a coinhibitory checkpoint on T cells [88]. The fusion protein is expected to be the fourth PD-1 neutralizing agent in clinical testing behind BMS' BMS-936558, Curetech's CT-011, and Merck's MK-3475.

In January 2011, Amgen acquired BioVex, which was developing OncoVex GM-CSF, a genetically modified herpes simplex virus 1 (HSV-1). The deal included $425 M upfront and $575 M of milestone payments. OncoVex GM-CSF is an oncolytic virus currently in phase III for the treatment of metastatic melanoma. The virus anti-cancer effect involves direct killing of tumor cells followed by immune activation that results from the virus immunogenicity and secretion of GM-CSF to the tumor microenvironment. In its phase II trial, OncoVex GM-CSF exhibited a unique clinical activity profile. Intratumor injection of the virus resulted in tumor shrinkage of injected as well as noninjected lesions. Responses were durable in a substantial portion of patients and overall survival was encouraging. An ongoing phase III trial is expected to generate results in 2012, using a primary endpoint of objective response lasting 6 months or more. Another phase III trial in head and neck cancer has been terminated in 2011.

In July 2011, BMS licensed IPH2102, an antibody targeting KIR receptors, from Innate Pharma. By binding the inhibitory KIR receptors on NK cells, the antibody, currently in phase I, is expected to promote an innate immune response against cancer cells. The deal included an upfront payment of $35 M as well as $430 M in development and commercialization milestones.

In September 2011, Bristol-Myers Squibb acquired ex-US commercialization rights (except in Japan, Korea, and Taiwan) for BMS-936558 from Japan-based Ono Pharmaceuticals. BMS-936558 is a fully human antibody targeting PD-1, for which BMS had originally held US rights. In return, Ono received certain commercialization rights for abatacept (Fc-fused CTLA4) in Japan. BMS-936558 is the most advanced PD-1 inhibitor in clinical testing, currently studied in melanoma, lung, and renal cancer. Initial results with this antibody as a single agent are encouraging [89].

In October 2011, MedImmune (the biologics arm of AstraZeneca) in-licensed two programs in the field of cancer immunotherapy. One deal involved licensing tremelimumab, an anti-CTLA4 antibody from Pfizer. MeDimmune assumed global development rights for Tremelimumab, which failed a phase III trial in melanoma in 2008. Future development will likely be based on pharmacodynamic biomarkers identified retrospectively in the failed phase III study. Pfizer retained the rights to use drug with specified types of combination therapies. Terms of the agreement were not disclosed. A second deal was signed with Portland-based AgonOx, which is developing OX40 agonists for the treatment of cancer. AgonOx is developing Fc-fused OX40 ligand as well as agonist antibodies. A murine antibody against OX40 led to immune activation and tumor shrinkage in a phase I trial (company's web site).

In October 2011, Genesis Biopharma announced a deal with the NIH for patents covering TIL therapy. The deal included an upfront payment of $1.2 M as well as undisclosed milestone payments and royalties. Following the deal, Genesis intends to turn the autologous cell-based treatment, which until now has been given as a service in medical centers, into a commercially available product. The company will offer the treatment, rebranded as Contigo, via several medical centers in the US and plans to manufacture it at a central production facility. The anticipated cost per patient is $120 thousand, similar to that of ipilimumab.

## Figures and Tables

**Table 1:** Current clinical trials in melanoma using monoclonal antibodies.

Company	Antibody	Target	Function	Status
	Antagonistic Abs.			
Bristol-Myers Squibb	Ipilimumab (Yervoy)	CTLA-4	Relieve immune block	Approved
Bristol-Myers Squibb	MDX-1106^1^	PD-1	Relieve immune block	Phase II (completed)
Curetech Ltd. (Israel)	CT-011	PD-1	Relieve immune block	Phase II (recruiting)
Merck	MK-3475	PD-1	Relieve immune block	Phase I (recruiting)
Bristol-Myers Squibb	MDX-1105-01	PD-L1	Relieve immune block	Phase I (recruiting)
	Agonistic Abs.			
Bristol-Myers Squibb	BMS-663513	4-1BB	Stimulate T cells	Phase II (completed)
Pfizer	CP870,893^2^	CD40	Stimulate T cells	Phase I (recruiting)
Tolerx	TRX518	GITR	Inhibit T regs	Phase I (on hold)
Portland Providence Medical Center	Anti-OX40	OX40	Stimulate T cells	Phase II (not open yet)

^1^Additional phase I studies are ongoing, in combination with Ipilimumab or with melanoma vaccines.

^2^Together with melanoma vaccine and an immune stimulant called Oncovir poly IC : LC (one phase I study) or with Tremelimumab (another phase I study).
